# Knowledge, Attitudes, Practices, and Factors Associated with Voluntary Blood Donation among University Students in Kilimanjaro, Tanzania

**DOI:** 10.1155/2016/8546803

**Published:** 2016-12-14

**Authors:** Elionora Elias, Wilhellmuss Mauka, Rune N. Philemon, Damian J. Damian, Michael J. Mahande, Sia E. Msuya

**Affiliations:** ^1^Institute of Public Health, Department of Community Medicine, Kilimanjaro Christian Medical University College (KCMUCo), P.O. Box 2240, Moshi, Tanzania; ^2^Institute of Public Health, Department of Epidemiology and Biostatistics, KCMUCo, Moshi, Tanzania; ^3^Northern Zone Blood Transfusion Centre, P.O. Box 823, Moshi, Tanzania; ^4^Department of Pediatrics, Kilimanjaro Christian Medical Centre (KCMC), Box 3010, Moshi, Tanzania; ^5^Department of Community Medicine, Kilimanjaro Christian Medical Centre (KCMC), Moshi, Tanzania

## Abstract

*Background*. Understanding the knowledge and awareness of blood donation among potential blood donors in the population, like young people, and the associated attitudes and practices is important.* Methodology*. This was a cross-sectional study whereby a self-administered questionnaire was used to collect information from the consenting participants.* Results*. A total of 422 participants were enrolled. Their mean age was 24.2 (SD 3.6) years. Of the 422, 30% have ever donated blood. 55% of those who had ever donated were repeated blood donors. Majority of the participants (93%) had positive attitudes towards blood donation and 88% were willing to donate in the future. Factors that were significantly associated with ever donating blood were male gender, knowing a person who has donated blood, knowledge of the amount of blood donated, willingness to donate in the future, and not expecting any postdonation reward.* Discussion*. High awareness, positive attitude, and high intention to donate in the future should be used to underscore the need to educate the young people on the value of blood donation in saving lives and to give them correct information on overall requirements for blood donation.

## 1. Introduction

Blood transfusion is a key component in modern health care in saving the lives of many people in routine and emergency situations like in gynecological conditions, pregnancy and childbirth, severe childhood illness, trauma and cancers, or medical hematological conditions [1]. The WHO recommends that, for any country to meet the minimum demand for blood, collection should be at least from 1% of the population [2]. On average, high income countries have 9 times higher donation rate compared to low income countries [3]. According to a study conducted in 2014 among the East African countries, Uganda had the highest blood collection units per a population of 1000 people (6.2) followed by Kenya (4.1) and Tanzania (3.6) [4] despite the fact that Tanzania had the highest population base among the three countries [5]. Notwithstanding the country variations, the overall rate of collection remained below the WHO minimum target of 10 units per a population of 1,000 people per year.

According to the Melbourne Declaration, voluntary nonremunerated blood donation (VNRBD) has been universally declared to be the cornerstone of safe blood [1]. In Tanzania, more than 80% are VNRBD and are from secondary schools (NBTS 2014 unpublished report). A study on blood donors revealed that the majority were aged between 24 and 35 years whereby more than three-quarters were male and in secondary education which may imply that the majority may have donated while attending secondary school [6]. However, it has been reported that college students also can be a very good source of quick and accessible quality blood, if they are motivated and recruited well as potential voluntary blood donors [7–9].

More than 50% of the population in sub-Saharan countries in Africa, including Tanzania, are aged between 15 and 64 years [10, 11]. More than 50% of Tanzanians are below 30 years of age [12] whereby the majority of potential blood donors are aged between 24 and 35 years [6, 13]. Acceptable age for blood donation in Tanzania is between 18 and 65 years. However, studies from elsewhere have shown that more than two-thirds of college students reported to have ever donated blood with varying reasons but many do not donate any longer [8, 14]. Some of the reasons given were tight schedule at college, lack of knowledge and awareness of blood donation [8], fear of needle or infection [15], and lack of opportunity [9]. There is no current published information on factors influencing voluntary blood donation among young people and among college students in Tanzania [16]. To fill that information gap, the study aimed at determining awareness, level of knowledge, and attitudes towards voluntary blood donation among university students by using the case of Kilimanjaro region. Such information would be vital in planning for raising awareness and helping young people to donate blood in the country.

## 2. Methods

### 2.1. Study Design and Area

This was a cross-sectional study that was conducted from April to June 2016 among university students of Kilimanjaro region. Kilimanjaro region is situated in Northern Tanzania and it is subdivided into seven districts, namely, Moshi urban, Moshi rural, Rombo, Mwanga, Same, Hai, and Siha. The main economic activities are food and cash crop production, commercial activities, tourism, and forestry. The region has the second lowest illiteracy rate (10%) in the country as well as high rate (94%) of school enrollment [17]. There are a total of four registered universities in the region which are Kilimanjaro Christian Medical University College (KCMUCo), Mwenge University College of Education (MWUCE), Stefano Moshi Memorial University College (SMMUCo), and Moshi University College (MUCo). KCMUCo and MUCo are located in Moshi urban district. KCMUCo trains health professionals of different cadres while MUCo offers cooperative and business education. SMMUCo and MWUCE are located in Moshi rural district. SMMUCo trains people in business administration, arts, and education whereas Mwenge University primarily offers bachelor and master's degrees in education [18]. However, there are other colleges in the region that offer nondegree programs like certificate and diploma level of education. Such colleges were not included lest to be underrepresented in this study.

The number of students at KCMUCo was 1791 (male = 1206, female = 585), at Mwenge University was 3360 (male = 2601, female = 759), at Moshi University was 3438 (male = 2623, female = 815), and at SMMUCo was 427 (male = 649, female = 135).

### 2.2. Study Population, Sample Size, and Sampling Procedures

The study population included students at the four selected universities who were aged 18 years and above. We excluded visiting students and those who did not consent to participate. Sample size was calculated by using Kish and Lisle formula for cross-sectional studies. The proportion of blood donation of 50% was used [19], with significance of 5% and power of 80%, and we added 10% of nonresponse. Substituting the values, the minimum sample size required was 422. A total of 460 university students from the four institutions were approached and invited to participate. Four hundred and twenty-two (422) agreed to participate, giving a response rate of 92%. Of 38 who refused to participate, 24 reported that they were busy and did not have time to participate and 14 did not participate because they reported they had no interest in the study and they did not want to give information.

Proportionate size sampling was used to get the total number of participants from each college. Among the 422 required participants, we anticipated to enroll 38% from Moshi University College (MUCo), 37% from Mwenge University, 20% from KCMUCo, and 5% from Stefano Moshi Memorial University College. At the university level, due to challenges of creating students' list, groups of students were approached and asked to participate in the study after brief introduction of the researchers and study objective. When the number (percent) of participants in a particular college was reached, the questionnaires were not distributed any further.

### 2.3. Data Collection Tools and Procedures

Questionnaires were used as a tool of data collection and were a self-administrative paper-pencil questionnaire. The filled questionnaires were collected by hand by the investigators and research assistants on the same day after distributing them to the students. The questionnaire was developed from review from previous studies [6, 8, 20, 21] and was piloted to 10 students prior to the study and validated. It had six parts. The first part collected information on sociodemographic characteristics of participants and had 5 questions. The second part was on the awareness and knowledge of blood donation with 5 questions [15, 22]. The third part was on practice of blood donation with 12 questions [22]. The fourth part was on attitudes towards blood donation with 10 questions based on “yes/no” answers, and the fifth part was on attitude towards blood donation and had 7 questions. The sixth part was on the source of information with 5 questions and the last part was on reasons for donating and not donating blood with two open ended questions.

### 2.4. Data Processing and Analysis

Questionnaires were organized and checked every day at the field for errors and to ensure their completeness. Data were entered and analyzed using the Statistical Package for the Social Sciences (SPSS) version 20. The first step was data cleaning by running frequency of each variable. Any information which was not clear was rechecked in the questionnaires. Responses from open ended questions were first categorized into themes and coded into categorical responses. Descriptive analysis was done for data summarization. For continuous variables, means with their respective measures of dispersion were used, while proportions were used for categorical data. Inferential statistics were carried out and strength of association was calculated by employing crude odds ratio (COR) through bivariate regression analysis which included all significant variables associated with blood donation practice on inferential statistics. Adjusted odds ratio (AOR) was carried out through backward binary regression analysis to determine factors significantly predicting blood donation involved in all significant variables from COR which were added simultaneously, and *p* value <0.05 was taken as statistically significant.

### 2.5. Ethical Clearance

Ethical clearance was sought from the KCMUCo ethical committee before starting the research. Permission to conduct research was sought from the Provosts of KCMUCo, MWUCE, SMMUCO, and MUCO. Written informed consent was obtained from all participants after explaining the aim of the study before administering the questionnaires. Confidentiality was maintained whereby numbers were used in the questionnaires instead of respondents' names for the purpose of gathering information.

## 3. Results

### 3.1. Sociodemographic Characteristics of the Participants

In the 422 participants, their age ranged from 18 to 44 years with mean age of 24.2 (SD 3.5) years and the majority, 287 (68%), were aged between 18 and 24 years. Of the 422, a large proportion of participants, 41.5% (175), were recruited from Moshi University College (MUCo), followed by 32.7% (138) from Mwenge University College of Education (MWENGE) and then 20.9% (88) from Kilimanjaro Christian Medical University College (KCMUCo) and the least 5.0% (21) from Stefano Moshi Memorial University College (SMMUCo). More than two-thirds of the participants were males (304, 72%) and single (347, 82%) and were taking bachelor's or master's degree program (359, 85%). Other characteristics are shown in Table 1.

### 3.2. Proportion of Blood Donation among University Students

Table 2 shows that, out of the 422 participants, 30% (126) had at least once donated blood in their lifetime. Of the 126 who had ever donated, more than half (55%) had donated twice or more. Majority reported a reason for their donation to be out of voluntarism (90.5%, 114); and 2 (1.6%) donated because they wanted to know their HIV health status, while sixty-nine percent stated that they had good experience from their last donation. More than ninety-six percent were willing to donate blood again in the future, and, in general, 83% (350) of all the students were willing to donate blood in the future irrespective of donation status before. Among (39) those who reported negative experience, 94.9% reported feeling dizziness on donation. Of the 296 participants who had never donated blood, 36% (152) did not donate blood because of lack of knowledge of blood donation and 13.2% (56) feared pain of needle prick. Other reasons are shown in Figure 2.

**Figure 1:**
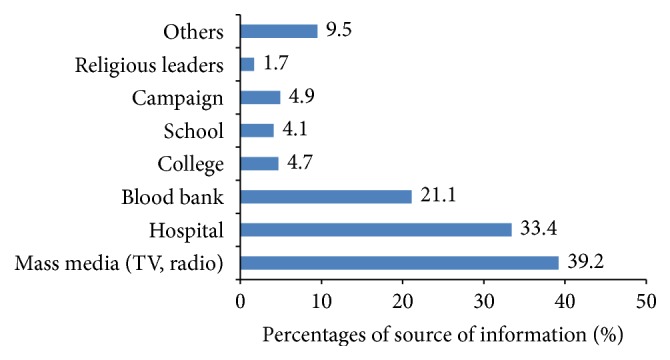
Source of information on blood donation. Total percentage exceeds 100% as some participants had more than one response.

**Figure 2:**
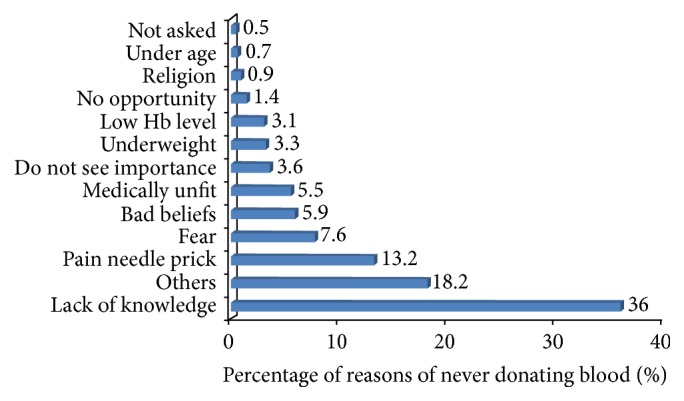
Reasons for never donating blood.

Media like radio and television (39.2%) followed by hospital (33.4%) and blood bank (21.1%) were the three most common sources of information on blood donation among the students who have heard or seen an advert concerning voluntary blood donation (see Figure 1).

### 3.3. Factors Associated with Blood Donation Practices

#### 3.3.1. Sociodemographic Characteristics

There was an association between gender of participants and blood donation practices whereby more than three-quarters of males had donated blood at least once (*p* = 0.004). The other variables were nonsignificantly associated with blood donation practice. They included age, program undertaken at the college, college, and the ethnic group.

#### 3.3.2. Awareness and Knowledge of University Students on Voluntary Blood Donation

There was a significant association between knowing a person who had donated blood and being aware of their blood group with blood donation, whereby 85.3% (360) knew someone who was a blood donor (*p* < 0.0001) and sixty-four percent (268) were aware of their blood group (*p* < 0.0001), respectively. About 37% of the 422 participants knew the amount of blood which could be donated at one setting of blood donation. Among those who had donated blood (126), almost half of them (49.6%) were knowledgeable about the amount that could be donated (*p* < 0.0001). Other variables such as seen or heard advert on blood donation, suitable age and weight, knowing of the frequency of donation a person can make per year, and screening transfusion transmissible infections before transfusion were not significantly associated with donation practices (Table 3).

#### 3.3.3. Attitude of Students on Voluntary Blood Donation

Attitudes of university students in Kilimanjaro on voluntary blood donation were positive in 94.7% of the university students who participated in the study (*p* = 0.023) (Table 3). Of the 422 students, 89.3% were willing to voluntarily donate blood to anyone (*p* = 0.003), 94.5% were willing to donate for a relative in need of blood (*p* = 0.023), and 81.4% did not expect reward for blood donation (*p* = 0.001). Willing to donate without knowing the religion of the recipient, suggestion on the voluntary donation to be the best form of blood donation, and considering blood donation as a noble act were not significantly associated with donation practice (Table 3).

In multivariate analysis, factors which remained significant predictors of blood donation were the following: males had 48% lower odds of donating blood compared to females (AOR (95% CI): 0.52 (0.03–0.92); *p* = 0.024). Those who were aware of their blood group had more than 11 times the odds of donating blood compared to those who had no knowledge of that (AOR (95% CI): 11.5 (5.5–23.9); *p* < 0.0001). Those who were willing to donate blood to anyone had 2.9 times the odds of donating blood compared with those who were not willing (AOR (95% CI): 2.9 (1.03–8); *p* < 0.044). Expecting postdonation rewards had 70% lower odds of donating blood compared to those who did not report expecting any reward (AOR (95% CI): 0.3 (0.16–0.7); *p* < 0.004) (Table 4).

## 4. Discussion

The results of this study show that there is low proportion of blood donors among the university students in Kilimanjaro, Tanzania. Student proportion of repeated blood donation among those who had ever donated was convincing. Awareness was seen to be high from participants such that a significant number reported to have heard about blood donation, knowing a person who had donated blood, and awareness of their blood groups. The participants had significant knowledge on the amount of blood to be donated in one setting. There was a significant positive attitude towards blood donation as the majorities were willing to donate in the future for anyone and did not expect any postdonation reward. Factors that were independently associated with ever donating blood were male gender, knowing a person who had ever donated blood, knowledge of the amount of blood donated, willingness to donate in the future, and not expecting reward for blood donation.

In this setting, 30% of the university students had ever donated blood. This proportion is higher than in other reports from African universities. Two studies in Ethiopia reported that 5 to 23% of the students at two different universities have ever donated blood [19, 23] while in Nigeria the proportion was 15% [8]. But this proportion of blood donation is low compared to the results from university students in Nepal (43%) or USA (56%), respectively [24, 25]. The difference from our setting with latter proportions might be due to the fact that blood donation activities in the country are highly dependent on secondary schools due to the easiness in organizing logistics.

What was positive about university students in Kilimanjaro was that more than 90% have positive attitude towards voluntary blood donation which was higher compared to a study in Ethiopia (47%) [26] and was almost as equal to other studies done in other universities [7, 27]. This high positive attitude may be due to methods of participants that were selected based on volunteerism, which are similar to ones done by Sabu and Bharatwaj and their colleagues, whereby in Ethiopia participants were selected based on checklist random sampling.

Ninety-five percent of those who have ever donated and 85% of those who had never donated blood are willing to donate in the future. These findings are aligning with other studies elsewhere [8, 27]. The National Blood Transfusion Services (NTBS) should use this opportunity and come with tailored strategies to target this group in order to increase their proportion of voluntary blood donation.

The main activity that should be prioritized is to increase information and education messages to university students and packages with simple and short messages to address knowledge gaps identified in this study. Many (more than a third) of those who had never donated cited lack of education on blood donation as a key reason for not donating blood. Similar findings have been reported in studies conducted in USA, India, Pakistan, Iran, Ethiopia, and Nigeria where in those studies it was also found that many students did not donate blood because of lack of enough knowledge [8, 22, 24–26]. Other students reported that they have never been asked to donate blood, which is why they have never donated, similar to observations in Pakistan and Nigeria [15, 28]. Maintaining an adequate and safe blood supply is a key concern among health planners in order to save lives and avert morbidity. Therefore, different models of delivering information to this group of young people who are key and potential blood donors in the country should be developed.

In the current study, being a male student increases the chance of donating blood compared with the female students. Studies from Iran, Nepal, and Nigeria among university students also reported that males donated more compared to females [8, 25]. It may be that females mistakenly believe they do not have adequate hemoglobin to donate blood due to menses or many are among the group that had no previous knowledge of blood transfusion [29]. There is a need to have qualitative studies to explore why women do not donate blood as males in this setting. Further, programs that are targeting youths or students with awareness and knowledge campaigns should think about specific messages that target women.

Willingness to donate to a relative or anyone without expecting financial rewards was a factor significantly associated with an increased chance of blood donation. A study conducted in Serbia among medical students showed that those who were willing to donate blood to anyone had higher odds of donating blood [30]. This type of altruistic behavior needs to be cultivated as it has been shown to influence voluntary blood donation and retention of blood donors [31–34]. This was contrary to the results observed in Pakistan where 58% of the students had negative attitude towards blood donation [9].

### 4.1. Strength and Limitations of the Study

This is the first study in Tanzania that sought to provide data on proportion and factors associated with blood donation among university students. The data is important for the directorate of the National Blood Transfusion Services and the Ministry of Health as it will enlighten challenges or barriers in addressing the increase of voluntary and nonremunerated blood donors among university students.

The study had some limitations. The sampling of the participants in all of the four universities in the region was a difficult task. Precaution should be taken in generalizing the results to other institutions in the region as there is a possibility of voluntary bias.

This was a cross-sectional study. Though several associations were observed with voluntary blood donation among university students in Kilimanjaro region, we cannot determine the temporal relationship between them. Information on ever or repeated blood donation relied on reports by the university students themselves and was not corroborated with any medical or registry reports. Hence, reporting bias could not be ruled out which might have an effect on the results towards estimating the proportion if the students reported the practice more than actual practice.

## 5. Conclusion

The study revealed that almost one-third of the university students in Kilimanjaro had ever donated blood with the majority of them willing to donate in the future. High awareness on blood donation and positive attitude should be taken as an opportunity to give these young and potential blood donors correct information on the process of blood donation and on frequency of blood donation as well as the knowledge on the value of blood in saving lives. Different models should be developed, piloted, and tested in the universities' community and people should be encouraged to donate blood voluntarily and regularly so as to achieve the WHO goal of 100% voluntary nonremunerated blood donation by 2020. Finally, female-tailored education messages should be developed to encourage more female students to donate blood in the university settings.

## Figures and Tables

**Table 1:** Sociodemographic characteristics of participants (*N* = 422).

Characteristics	*n* (%)^*∗*^	Percentage (%)
*Age (years), mean (SD)*	24 (3.5)	
*Age, category (years)*		
18–24	287	68
25–34	124	29.4
35–44	11	2.6
*Sex*		
Female	118	28
Male	304	72
*Marital status*		
Married	36	8.5
Single	347	81.5
Other	42	10
*Name of university*		
MUCo	175	41.5
MWENGE	138	32.7
KCMUCo	88	20.9
SMMUCo	21	5
*Program taken*		
Certificate	8	1.9
Diploma	46	10.9
Degree	359	85.1
Masters	9	2.1
*Tribe*		
Chagga	127	30.1
Nyamwezi	50	11.8
Sukuma	50	11.8
Others	195	46.1

^**∗**^Column percentage.  SD: standard deviation.

**Table 2:** Practice of university students on blood donation among blood donors (*N* = 422).

Characteristics	Frequency	Percentage
*Ever donated blood*	126	29.9
*Frequency of donation in lifetime *(*N* = 126)		
Once	57	45.2
Repeated	69	54.8
*Donated in the past 5 years*	124	98.4
*Donated in the past 12 months*	123	97.6
*Types of donation*		
Voluntary	114	90.5
Paid	2	1.6
Replacement	10	7.9
*Main reason(s) for the last time donation*		
Voluntarism	114	90.5
HIV results	2	1.6
Replacement	10	7.9
*Would you donate blood again in the future?*		
Yes	120	95.2
No	6	4.8
*The place of your last donation*		
Blood Transfusion Centre	15	12
Hospital	28	22.4
School	82	65.6
*Experienced negative effect after donation*		
Yes	39	31
No	87	69
*Postdonation negative effect *(*N* = 39)		
Pain	1	1
Dizziness	37	94.9
Fever	1	2.6

**Table 3:** Association between participants' sociodemographic characteristics, awareness, knowledge, and blood donation practice (*N* = 422).

Characteristics^*∗*^	Ever donated blood^*∗∗*^		
	*N* (%)	*n* (%)	*p* value
*Sociodemographic*			
Sex			0.004
Female	118 (28)	23 (18.3)	
Male	304 (72)	103 (81.7)	
*Awareness of blood donation*			
Ever heard of blood donation			0.002
Yes	389 (92.2)	124 (31.9)	
No	33 (7.8)	2 (6.1)	
Knew a person who had donated blood			<0.0001
Yes	360 (85.3)	120 (95.2)	
No	62 (14.7)	6 (4.8)	
Awareness of blood group			<0.0001
Yes	268 (64)	116 (92.8)	
No	150 (35.8)	8 (6.4)	
*Knowledge of blood donation *			
Amount of blood donated per time			<0.004
Wrong answer	268 (63.5)	67 (53.2)	
Correct answer	154 (36.5)	59 (46.8)	
*Attitude towards blood donation*			
Attitude towards blood donation			0.028
Positive	392 (94.7)	122 (98.4)	
Negative	22 (5.3)	2 (1.6)	
Are you willing to donate blood to a relative?			0.023
Yes	399 (94.5)	124 (98.4)	
No	23 (5.5)	2 (1.6)	
Are you willing to donate blood to anyone?			0.004
Yes	377 (89.3)	121 (96)	
No	45 (10.7)	5 (4)	
Expectation for reward after donation			0.001
Yes	77 (18.6)	11 (8.9)	
No	337 (81.4)	113 (91.1)	

^*∗∗*^Column percent.  ^*∗*^Variables include only significant association, *p* < 0.05.

**Table 4:** Factors associated with blood donation.

Characteristics	Ever donated blood			
	COR (95%CI)	*p* value	AOR (95% CI)^*∗*^	*p* value
*Sex*				
Female	1		1	
Male	2.1 (1.3–3.5)	0.004	0.52 (0.3–0.92)	0.024
*Awareness of blood group*				
No	1		1	
Yes	12 (5.9–24.6)	<0.001	11.5 (5.5–23.9)	<0.0001
*Amount of blood donated per time*				
Wrong answer	1		1	
Correct answer	2.06 (1.3–3.20)	0.001	1.5 (1.6–0.96)	**0.075**
*Attitude towards blood donation*				
Negative	1			
Positive	4.5 (1.0–19.6)	0.04	—	
*Are you willing to donate blood to a relative?*				
No	1		—	
Yes	4.7 (1.1–20.5)	0.038		
*Are you willing to donate blood to anyone?*				
No	1		1	
Yes	3.9 (1.5–9.8)	0.006	2.9 (1.03–8)	0.044
*Expectation for reward after donation*				
No	1		1	
Yes	0.3 (0.17–0.65)	0.001	0.3 (0.16–0.7)	0.004

^**∗**^ *COR (crude odds ratio)* involved bivariate regression that included all significant variable associated with blood donation practice; *AOR (adjusted odds ratio)* involved multiple regression of all significant variables from COR that were added simultaneously.
